# Transcriptomic, 16S ribosomal ribonucleic acid and network pharmacology analyses shed light on the anticoccidial mechanism of green tea polyphenols against *Eimeria tenella* infection in Wuliangshan black-boned chickens

**DOI:** 10.1186/s13071-023-05922-x

**Published:** 2023-09-19

**Authors:** Hai-Yang Song, Meng-Ling Deng, Jian-Fa Yang, Jun Ma, Fan-Fan Shu, Wen-Jie Cheng, Xing-Quan Zhu, Feng-Cai Zou, Jun-Jun He

**Affiliations:** 1 Faculty of Animal Science and Technology, Yunnan Agricultural University, Kunming, 650201 Yunnan People’s Republic of China; 2 Key Laboratory of Veterinary Public Health of Yunnan Province, College of Veterinary Medicine, Yunnan Agricultural University, Kunming, 650201 Yunnan People’s Republic of China; 3 Laboratory of Parasitic Diseases, College of Veterinary Medicine, Shanxi Agricultural University, Taigu, 030801 Shanxi People’s Republic of China

**Keywords:** *Eimeria tenella*, Green tea polyphenol, Wuliangshan black-boned chicken, RNA-seq, 16S rRNA, Network pharmacology

## Abstract

**Background:**

*Eimeria tenella* is an obligate intracellular parasitic protozoan that invades the chicken cecum and causes coccidiosis, which induces acute lesions and weight loss. Elucidating the anticoccidial mechanism of action of green tea polyphenols could aid the development of anticoccidial drugs and resolve the problem of drug resistance in *E. tenella*.

**Methods:**

We constructed a model of *E. tenella* infection in Wuliangshan black-boned chickens, an indigenous breed of Yunnan Province, China, to study the efficacy of green tea polyphenols against the infection. Alterations in gene expression and in the microbial flora in the cecum were analyzed by ribonucleic acid (RNA) sequencing and 16S ribosomal RNA (rRNA) sequencing. Quantitative real-time polymerase chain reaction was used to verify the host gene expression data obtained by RNA sequencing. Network pharmacology and molecular docking were used to clarify the interactions between the component green tea polyphenols and the targeted proteins; potential anticoccidial herbs were also analyzed.

**Results:**

Treatment with the green tea polyphenols led to a reduction in the lesion score and weight loss of the chickens induced by *E. tenella* infection. The expression of matrix metalloproteinase 7 (*MMP7*),* MMP1*, nitric oxide synthase 2 and ephrin type-A receptor 2 was significantly altered in the *E. tenella* infection plus green tea polyphenol-treated group and in the *E. tenella* infection group compared with the control group; these genes were also predicted targets of tea polyphenols. Furthermore, the tea polyphenol (-)-epigallocatechin gallate acted on most of the targets, and the molecular docking analysis showed that it has good affinity with interferon induced with helicase C domain 1 protein. 16S ribosomal RNA sequencing showed that the green tea polyphenols had a regulatory effect on changes in the fecal microbiota induced by *E. tenella* infection. In total, 171 herbs were predicted to act on two or three targets in MMP7, MMP1, nitric oxide synthase 2 and ephrin type-A receptor 2.

**Conclusions:**

Green tea polyphenols can directly or indirectly regulate host gene expression and alter the growth of microbiota. The results presented here shed light on the mechanism of action of green tea polyphenols against *E. tenella* infection in chickens, and have implications for the development of novel anticoccidial products.

**Graphical Abstract:**

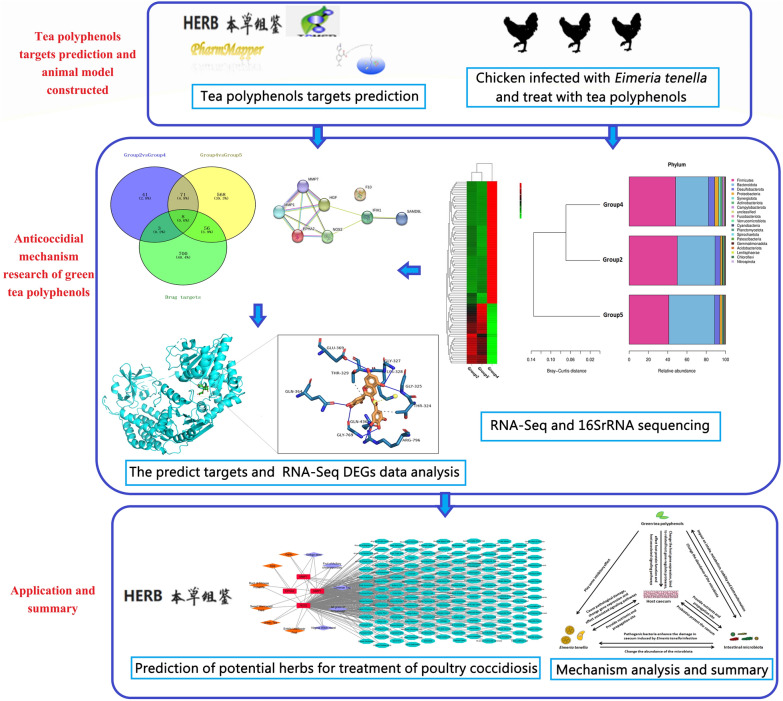

**Supplementary Information:**

The online version contains supplementary material available at 10.1186/s13071-023-05922-x.

## Background

The Wuliangshan black-boned chicken, which has meat of high quality and significant economic value, is an indigenous breed of Yunnan Province, southwestern China [1, 2]. Avian coccidiosis caused by infection with species of the genus *Eimeria* is one of the primary causes of economic loss in the chicken breeding industry, and *Eimeria tenella* is one of the most virulent species of this genus that infects chickens. The economic cost of prophylaxis and treatment for coccidiosis, and loss of productivity due to this disease in the global chicken population, was estimated to be £10.4 billion in 2016 [3]. Due to the extensive use of anticoccidial drugs, resistance to these is common in *Eimeria* species [4]. Thus, new drugs need to be developed to control avian coccidiosis, and some plant compounds show promise for this.

Green tea is one of the most popular hot drinks worldwide. The main components of green tea polyphenols are (-)-epicatechin (EC), (-)-epigallocatechin (EGC), (-)-epicatechin gallate (ECG), (-)-gallocatechin gallate (GCG) and (-)-epigallocatechin gallate (EGCG) [5–7]. EGCG has potent antioxidant effects, scavenging cellular reactive oxygen, inhibiting the formation of free radicals, and reducing lipid peroxidation [8]. Green tea polyphenols can influence digestion, absorption, metabolism and immunity [9–11]. Green tea and green tea polyphenols have been used to enhance the treatment effects of anticancer drugs [12, 13]. They are also used as nutritional supplements to promote health [14], as feed additives to improve productivity [15, 16], and for the treatment of disease in livestock [17, 18]. Tea has been shown to have anticoccidial effects, although the mechanism responsible for these has yet to be elucidated [19].

*Eimeria* spp. have a high degree of host specificity. The invasion of *Eimeria* spp. involves multiple interactions with the host cells, such as cell recognition, adhesion, and establishment of an intracellular niche [20]. *Eimeria* infection can disrupt the gut microbial communities of the host, promoting pathogen proliferation and influencing nutrient absorption [21]. Thus, it is important to elucidate the changes in the host’s gene expression and in its enteric microorganisms during infection with *E. tenella*. Ribonucleic acid (RNA) sequencing (RNA-seq), a popular method for the analysis of gene expression, can be used to establish a reference dataset of gene expression for disease diagnosis [22] and vaccine and drug development [23, 24]. 16S ribosomal RNA (rRNA) sequencing is useful for understanding shifts in the gut microbial community at different biological statuses [25].

Infection with *Eimeria* can result in cellular destruction in the host, which is coordinated by multiple genes and their proteins [26]. Traditional Chinese medicine employs multiple compounds with multiple targets to improve efficacy compared to the use of single-target drugs [27, 28]. Network pharmacology can be used to construct a compound-protein/gene-disease network to predict complex drug-target interactions [29, 30], and has been widely used in research on viruses [31], bacteria [32] and parasites [33].

We constructed a model of *E. tenella* infection in Wuliangshan black-boned chickens and compared the anticoccidial capacity of green tea polyphenols at different doses. RNA-seq, network pharmacology and 16S rRNA sequencing were performed to reveal the expressional changes of host genes and the alterations of the gut microbiota during *E. tenella* infection with green tea polyphenols treament, and to predict other types of anticoccidial herbs. This work sheds light on the potential anticoccidial mechanism and regulation of microorganisms by green tea polyphenols, which may be beneficial for the development of anticoccidial drugs, vaccines and feed additives.

## Methods

### Animal experiment and sample collection

A total of 75 Wuliangshan black-boned chickens (7 days old) were purchased from a local poultry hatchery. The chickens were randomly divided into five groups, with 15 chickens in each group. Green tea polyphenols were purchased from Anhui Redstar Pharmaceutical (China). The chickens in groups 1–3 were administered green tea polyphenols (GTPs) orally at the following doses: 50 mg GTPs kg^−1^ body weight (group 1), 200 mg GTPs kg^−1^ body weight (group 2), and 350 mg GTPs kg^−1^ body weight (group 3). The chickens in group 4 were not treated with any tea polyphenols, and the chickens in group 5 comprised the blank control group. The chickens were treated with green tea polyphenols once a day from the age of 8 days until they were 28 days old. The chickens in groups 1–4 were orally infected with 50,000 sporulated *E. tenella* oocysts at 21 days of age. All of the procedures involving animals were approved by the Animal Ethics Committee of Yunnan Agricultural University (permit no. 202110009).

All of the chicken were sacrificed at 28 days of age for the collection of cecal tissue and cecal content samples, which were stored in liquid nitrogen until RNA and DNA extraction. Feces were collected from chickens at 6–8 days post-infection with *E. tenella* oocysts, and the number of oocysts per gram was calculated by using the McMaster method [34]. The oocyst index and lesion scores were evaluated following previously published methods [35, 36]. Relative weight gain rate was calculated as (average body weight gain of each group/average body weight gain of the untreated and unchallenged control group) × 100%, and anticoccidial index (ACI) was calculated as (survival rate + relative weight gain) − (lesion score + oocyst index). ACI < 120 were considered to indicate no anticoccidial activity, ACI of 120–160 to indicate partial anticoccidial efficiency, and ACI > 160 to indicate high anticoccidial efficiency.

### Analysis of the putative targets of green tea polyphenols

The putative targets of green tea polyphenols were obtained from the High-throughput Experiment- and Reference-guided Database of Traditional Chinese Medicine (HERB), the Traditional Chinese Medicine Systems Pharmacology Database and Analysis Platform (TCMSP) and PharmMapper databases. (-)-Epicatechin (EC), (-)-gallocatechin gallate (GCG), (-)-epigallocatechin (EGC), (-)-epicatechin gallate (ECG) and (-)-epigallocatechin gallate (EGCG) were used as the search terms for ingredients or chemical names in the HERB and TCMSP databases to search for target genes. The three-dimensional structures of EC, GCG, EGC, ECG and EGCG were downloaded from PubChem and saved in SDF format which were used to calculate and find genes in PharmMapper. We used these data to search for proteins in the STRING database; duplicate data were removed.

### RNA extraction, library construction, RNA-seq and data analyses

Three samples were randomly selected from group 2 [*E. tenella* infection (ET) plus green tea polyphenol treatment (GTP) group], group 4 (ET group) and group 5 (control group). Total RNA was extracted by using Trizol reagent (15596018; Thermo Fisher Scientific) following the manufacturer’s instructions. The total quantity of RNA and its purity were analyzed by using a Bioanalyzer 2100 and RNA 6000 Nano LabChip Kit (5067–1511; Agilent, CA). High-quality RNA samples with a RNA integrity number > 7.0 were used for the construction of a high-throughput sequencing library. After the treatment of the uracil-labeled double-stranded DNA with heat-labile uracil-DNA glycosylase (New England Biolabs), the ligated products were amplified by polymerase chain reaction (PCR) as follows: initial denaturation at 95℃ for 3 min; eight cycles of denaturation at 98 ℃ for 15 s, annealing at 60 ℃ for 15 s, and extension at 72 ℃ for 30 s; final extension was performed at 72 ℃ for 5 min; 2× 150-base pair paired-end sequencing (PE150) of the final complementary DNA (cDNA) library was performed on an Illumina Novaseq 6000, and the sequencing quality was assessed using Q20, Q30 and GC content. We aligned reads of all samples to the chicken reference genome (https://ftp.ensembl.org/pub/release-109/fasta/gallus_gallus/) using the HISAT2 package. The transcript was assembled with mapped reads of each sample by StringTie software with default parameters. Then, the transcripts of all samples were merged to reconstruct a comprehensive transcriptome by gffcompare software. After the final transcripts had been generated, StringTie and Ballgown were used to estimate the expression abundance of all transcripts by calculating the fragments per kilobase per million mapped fragments. Gene differential expression analysis was performed by using DESeq2 software. Genes with a false discovery rate (*q*-value) < 0.05 and absolute fold change ≥ 2 were considered to be differentially expressed genes (DEGs). DEGs were then subjected to enrichment analysis using Gene Ontology (GO) functions and pathway enrichment analysis (Kyoto Encyclopedia of Genes and Genomes; KEGG).

### Validation by quantitative real-time PCR

Eleven genes were randomly selected for gene expression verification by quantitative real-time PCR (qPCR). The PCR primers are listed in Additional file 1: Table S1. The RNA samples used for RNA-seq were used for first-strand complementary DNA (cDNA) synthesis by using TransScript Uni All-in-One First-Strand cDNA Synthesis SuperMix for qPCR (One-Step gDNA Removal) Kit (TransGen Biotech). The qPCR reactions were performed using the PerfectStart Green qPCR SuperMix Kit (TransGen Biotech) according to the manufacturer’s instructions. Glyceraldehyde-3-phosphate dehydrogenase was used as an internal control and relative gene expression was calculated using the 2^−ΔΔCt^ method.

### 16S rRNA sequencing and data analysis

The DNA of cecal contents corresponding to the RNA-seq caecum samples was extracted using a cetyltrimethylammonium-bromide kit (GuangZhou Chemical Reagent Factory, Guangdong, China) in accordance with the manufacturer’s instructions. PCR amplification was performed using V3-V4 primers. The PCR products that amplified with these primers were purified by using AMPure XT beads (Beckman Coulter Genomics, Danvers, MA), and quantified by Qubit (Invitrogen, USA). The abundance and quality of the amplicon library were assessed using an Agilent 2100 Bioanalyzer (Agilent, USA) and the Library Quantification Kit for Illumina (Kapa Biosciences, Woburn, MA), respectively. The 16S rRNA libraries were sequenced using the NovaSeq PE250 platform. Paired-end reads were assigned to samples based on their unique barcode and truncated by cutting off the barcode and primer sequence. Paired-end reads were merged using FLASH software. Quality filtering of the raw reads was performed by fqtrim (v0.94). Vsearch software (v2.3.4) was used for chimeric sequences filtering. The feature table and feature sequences were obtained after dereplication using DADA2. Alpha diversity and beta diversity were calculated by normalizing to the same sequences randomly. Feature abundance was normalized using SILVA classifier. Alpha diversity was used to analyze the complexity of species diversity through the use of three indexes: Chao1, Shannon, Simpson. Beta diversity was calculated with QIIME2. The Basic Local Alignment Search Tool was used for sequence alignment, and the feature sequences were annotated through the SILVA and NT-16S databases using representative sequences.

### Construction of a protein–protein interaction network, compound-target-KEGG network, molecular docking and herb-target network

The protein–protein interaction network of the obtained putative targets of tea polyphenols were analyzed using the STRING database, and a compound-target-KEGG network was constructed by Cytoscape (version 3.6.0). The crystal structures of proteins were downloaded from the Protein Data Bank (PDB). The two-dimensional structures of small molecules were downloaded from the PubChem database, and converted into a PDB file using OpenBabel 2.4.1 software. The analysis of low molecular weight ligands, protein receptors and molecular docking was performed using SailVina and Autodock, and the protein ligand interaction profiler web tool was used to identify the interactions. The results of docking were visualized by PyMOL software (version 2.3.4). The herb-target network was constructed by Cytoscape (version 3.6.0) to predict herbs that interacted with the important targets identified in this study. All of the information on the predicted herb-target interactions was obtained from the HERB database.

## Results

### Anticoccidial efficacy of green tea polyphenols

Treatment with green tea polyphenols reduced the lesion score and weight loss induced by *E. tenella* infection (Table 1). The lesion score in group 3 was the lowest of all the challenged groups. The rates of body weight gain of chickens in the green tea polyphenol groups were higher than that in the untreated challenged group.

**Table 1:** Anticoccidial index (*ACI*) of the infected chickens under the different doses of green tea polyphenols (*GTPs*)

Groups (GTP dose)	Dose of GTPs (mg/kg)	Challenge with *Eimeria tenella* sporulated oocysts at 21 days	Lesion score	Oocyst index	Relative weight gain (%)	Survival rate (%)	ACI
Group 1 (50 mg GTPs kg^−1^ body weight)	50	50,000	16.00	5	48.31	100	127.31
Group 2 (200 mg GTPs kg^−1^ body weight)	200	50,000	9.33	3.33	56.58	100	143.92
Group 3 (350 mg GTPs kg^−1^ body weight)	350	50,000	8.66	3.33	59.43	100	147.44
Group 4 (No GTPs administered)	0	50,000	13.30	6.66	35.64	100	115.68
Group 5 (Blank control)	0	0	0	0	100	100	200

### Tea polyphenol-associated targets

EC, EGC, ECG, GCG and EGCG were used as the key substances to search the databases, and 506, 87 and 261 drug protein targets were obtained from the HERB, TCMSP and PharmMapper databases, respectively. These data were converted according to* Gallus* origin in the STRING database. A total of 767 tea polyphenol-associated protein targets were obtained after removing the duplicate data. The details of these protein targets are listed in Additional file 2: Table S2.

### Screening and annotation of DEGs in the ceca of infected chickens

**Fig. 1:**
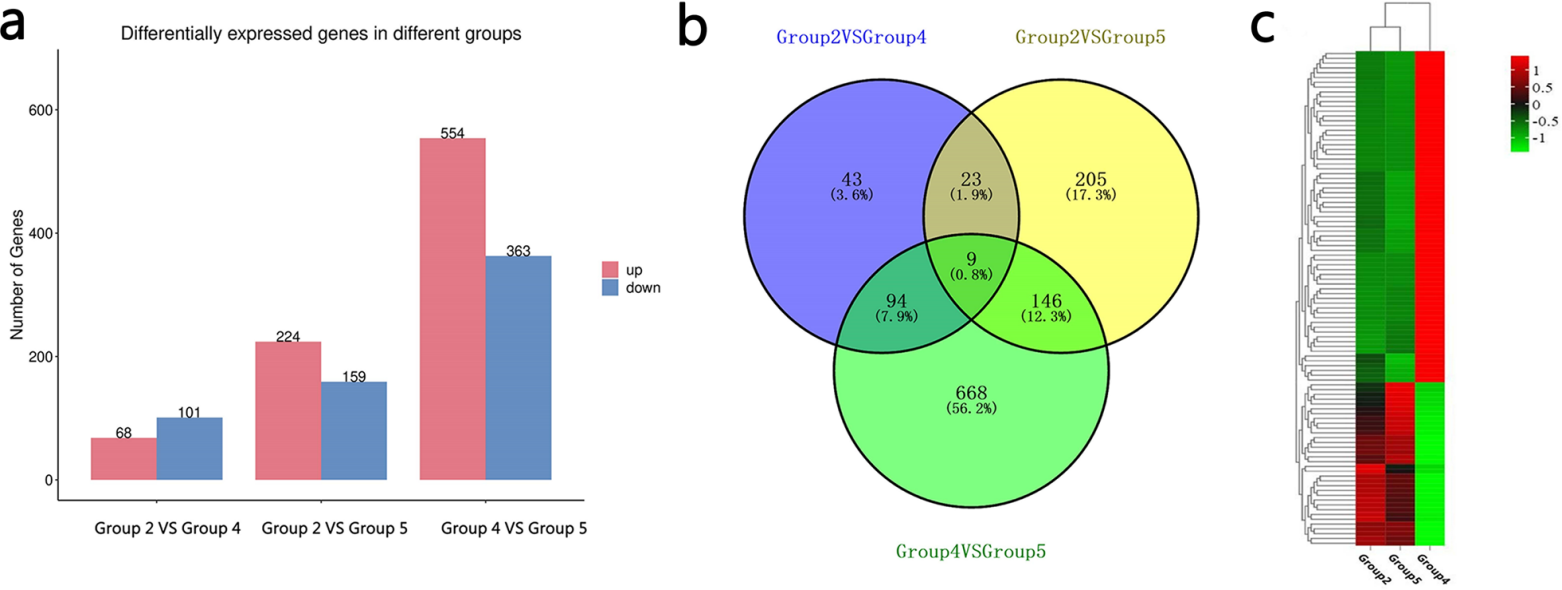
**a**–**c** Differentially expressed genes (DEGs) of the different groups. **a** Histogram showing significantly up- and downregulated genes between the different groups. **b** Venn diagram showing relationships between groups with respect to DEGs. **c** Heatmap of the common genes found between group 2 [*Eimeria tenella* challenge (ET) plus green tea polyphenol treatment (GTP) group; ET + GTP group] and group 4 (ET group), and between group 4 (ET group) and group 5 (control group)

A total of 68 significantly upregulated and 101 significantly downregulated genes were identified between group 2 (ET + GTP group) and group 4 (ET group); 224 significantly upregulated and 159 significantly downregulated genes between group 2 (ET + GTP group) and group 5 (control group); and 554 significantly upregulated and 363 significantly downregulated genes between group 4 (ET group) and group 5 (control group) (Fig. 1a). Intersection analysis showed that nine genes [matrix metalloproteinase 7 (*MMP7*),* MMP1*, nitric oxide synthase 2 (*NOS2*), ephrin type-A receptor 2 (*EPHA2*), secreted phosphoprotein 1 (*SPP1*), cell wall biogenesis 43 C-terminal homolog, dual-specificity phosphatase 6 (*DUSP6*), ENSGALG00000051623 and KCNJ5] related to enzyme and membrane proteins were common to the different groups (Fig. 1b). A total of 103 common DEGs were found between group 2 (ET + GTP group) and group 4 (ET group) and between group 4 (ET group) and group 5 (control group); the clustering heatmap of the common DEGs is shown in Fig. 1c.

### Network construction and molecular docking

**Fig. 2:**
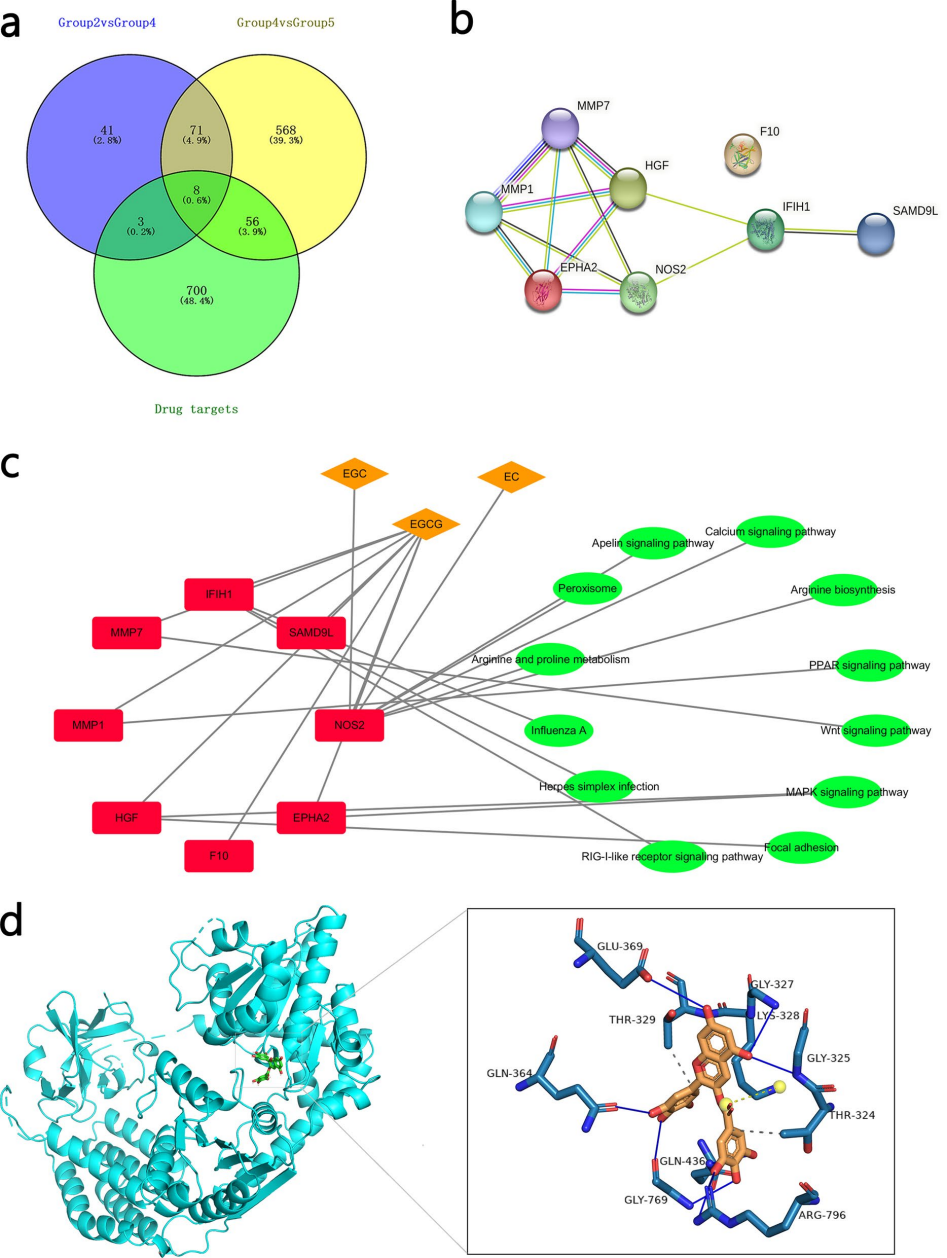
**a**–**d** Network construction and molecular docking. **a** Venn diagram showing predicted protein targets in group 2 (ET + GTP group) compared with group 4 (ET group), and in group 4 (ET group) compared with group 5 (control group). **b** Protein–protein interaction network of eight drug targets. **c** Compound-target-Kyoto Encyclopedia of Genes and Genomes (KEGG) network diagram. **d** Molecular docking of (-)-epigallocatechin gallate (*EGCG*) and interferon induced with helicase C domain 1 (*IFIH1*) protein. *MMP* Matrix metalloproteinase,* HGF* hepatocyte growth factor,* F10* coagulation factor X,* EPHA2* ephrin type-A receptor 2,* SAMD9L* sterile alpha motif domain containing 9 like,* NOS2* nitric oxide synthase 2,* EGC* (-)-epigallocatechin,* EC* (-)-epicatechin; for other abbreviations, see Fig. 1

We used the 767 associated protein targets of the tea polyphenols, 123 protein targets in group 2 (ET + GTP group) compared with group 4 (ET group) and 703 protein targets in group 4 (ET group) compared with group 5 (control group), to produce a Venn diagram (Fig. 2a). There were fewer proteins than RNA-seq gene data because for some genes the protein name was lacking, or in some cases proteins encoded by different genes had the same name. The Venn diagram showed eight proteins common to the groups: MMP7, MMP1, NOS2, EPHA2, sterile alpha motif domain containing 9 like, coagulation factor X, interferon induced with helicase C domain 1 (IFIH1) and hepatocyte growth factor. We used these eight protein targets to construct a protein–protein interaction network, which has seven nodes and 12 edges based on a minimum 0.15 interaction score (Fig. 2b). Three compounds and 12 KEGG pathways were used to construct the compound-target-KEGG network for these eight protein targets (Fig. 2c). The compound-target-KEGG network showed that EGCG targets all of these eight proteins, and that both EC and EGC target the NOS2 protein; NOS2 was the target of most of these KEGG pathways. No targets were identified for ECG or GCG (data not shown). The eight targets were of relevance to the peroxisome proliferator-activated receptor signaling pathway, wingless-related integration site signaling pathway, mitogen-activated protein kinase (MAPK) signaling pathway, focal adhesion, RIG-I-like receptor signaling pathway, herpes simplex infection, influenza A, arginine and proline metabolism, peroxisome, apelin signaling pathway, calcium signaling pathway and arginine biosynthesis.

We assessed the expected interaction between EGCG and IFIH1 because the crystal structure of only the latter protein of *Gallus* was found in the PDB database (Fig. 2d). Molecular docking revealed an interaction between IFIH1 and the small molecule EGCG. The protein ligand interaction profiler web tool showed that the amino acid residues THR-324, LYS-328 and THR-329 exhibited hydrophobic interactions with EGCG; that the amino acid residue GLY-325, GLY-327, GLN-364, GLU-369, GLN-436, GLY-769 and ARG-796 engaged in hydrogen bond interactions with EGCG; and that the amino acid residue LYS-328 showed salt bridges interactions with EGCG. The binding energy of the ligand molecule with the receptor was − 9.8 kcal/mol, which indicates good mutual affinity.

### Enrichment analyses of GO and the KEGG pathway

**Fig. 3:**
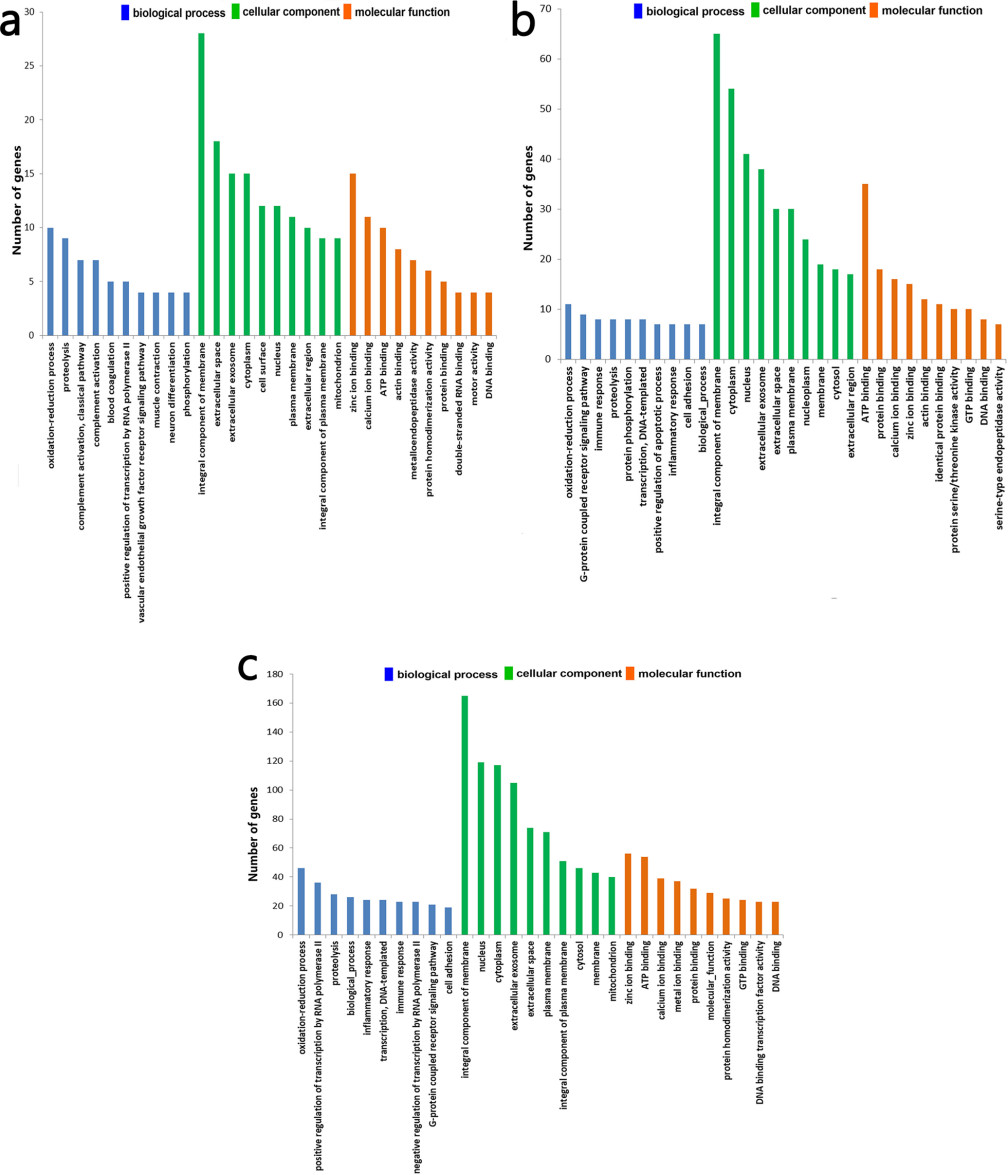
**a**–**c** Histogram of enriched Gene Ontology (GO) terms in the different groups. GO enrichment of DEGs between **a** group 2 (ET + GTP group) and group 4 (ET group), **b** group 2 (ET + GTP group) and group 5 (control group), and **c** group 4 (ET group) and group 5 (control group). For other abbreviations, see Fig. 1

**Fig. 4:**
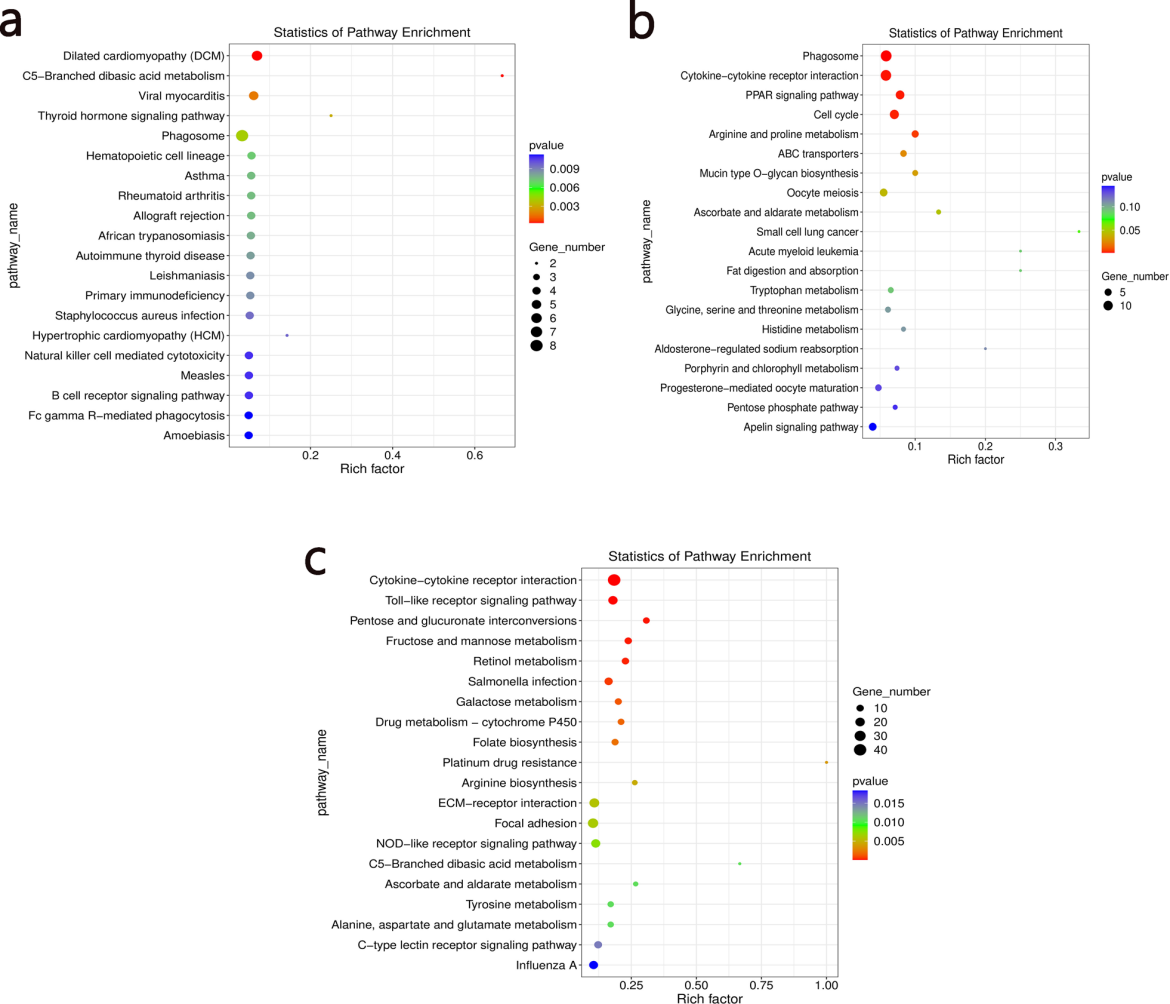
Bubble chart of the top 20 pathways identified by KEGG pathway enrichment analysis of DEGs between **a** group 2 (ET + GTP group) and group 4 (ET group), **b** group 2 (ET + GTP group) and group 5 (control group), and **c** group 4 (ET group) and group 5 (control group). For other abbreviations, see Figs. 1 and 2

The top 10 enriched GO terms (biological process, cellular component and molecular function) between different groups are shown in Fig. 3. GO enrichment showed common enrichment in oxidation–reduction process and proteolysis in biological process; integral component of membrane, nucleus and cytoplasm in cellular component; common enrichment in zinc ion binding, ATP binding and calcium ion binding in molecular function. The top 20 statistically significant pathways of the KEGG pathway enrichment analysis are shown in Fig. 4. The top five pathways enriched with DEGs between group 2 and group 4 were dilated cardiomyopathy, C5-branched dibasic acid metabolism, viral myocarditis, thyroid hormone signaling pathway and phagosome. The top five pathways enriched with DEGs between group 2 and group 5 were phagosome, cytokine-cytokine receptor interaction, peroxisome proliferator-activated receptor signaling pathway, cell cycle, arginine and proline metabolism. The top five pathways enriched with DEGs between group 4 and group 5 were cytokine-cytokine receptor interaction, Toll-like receptor signaling pathway, pentose and glucuronate interconversions, fructose and mannose metabolism and retinol metabolism.

### Validation of gene expression using qPCR

**Fig. 5:**
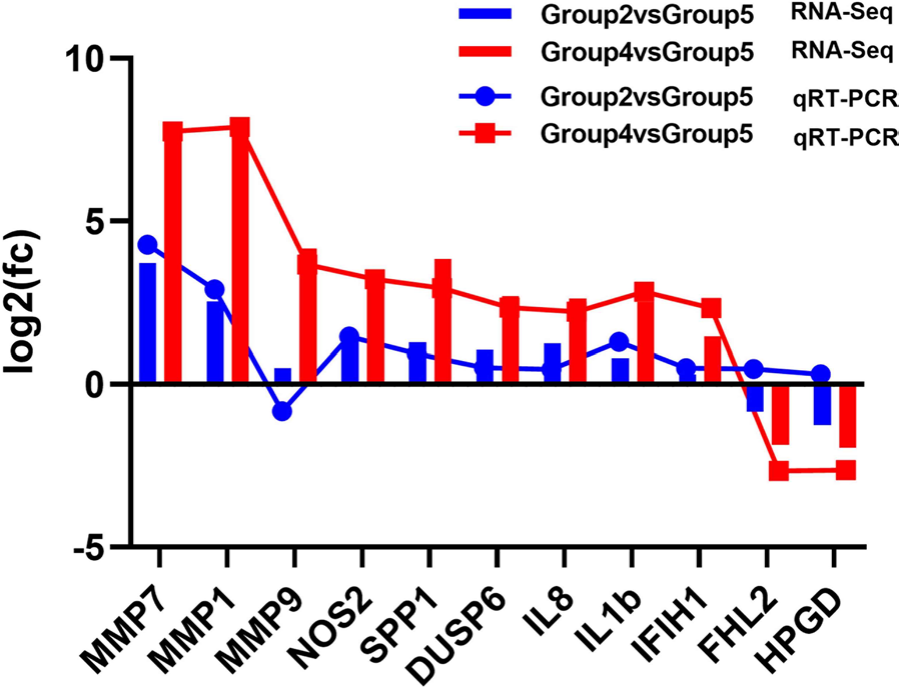
Changes in gene expression determined by quantitative real-time polymerase chain reaction (*qRT-PCR*) and ribonucleic acid (RNA) sequencing (*RNA-seq*). For other abbreviations, see Figs. 1 and 2

qPCR was performed to validate the host gene expression data from RNA-seq for 11 genes:* MMP7*,* MMP1*,* MMP9*,* NOS2*,* SPP1*, IL-8, IL1B,* DUSP6*,* IFIH1*, four and a half LIM domains 2 and 15-hydroxyprostaglandin dehydrogenase. The qPCR results (Fig. 5) were consistent with those of the RNA-seq, which confirmed that the RNA-seq data were reliable.

### Effects of *E. tenella* infection and green tea polyphenol treatment on chicken gut microbiota

**Fig. 6:**
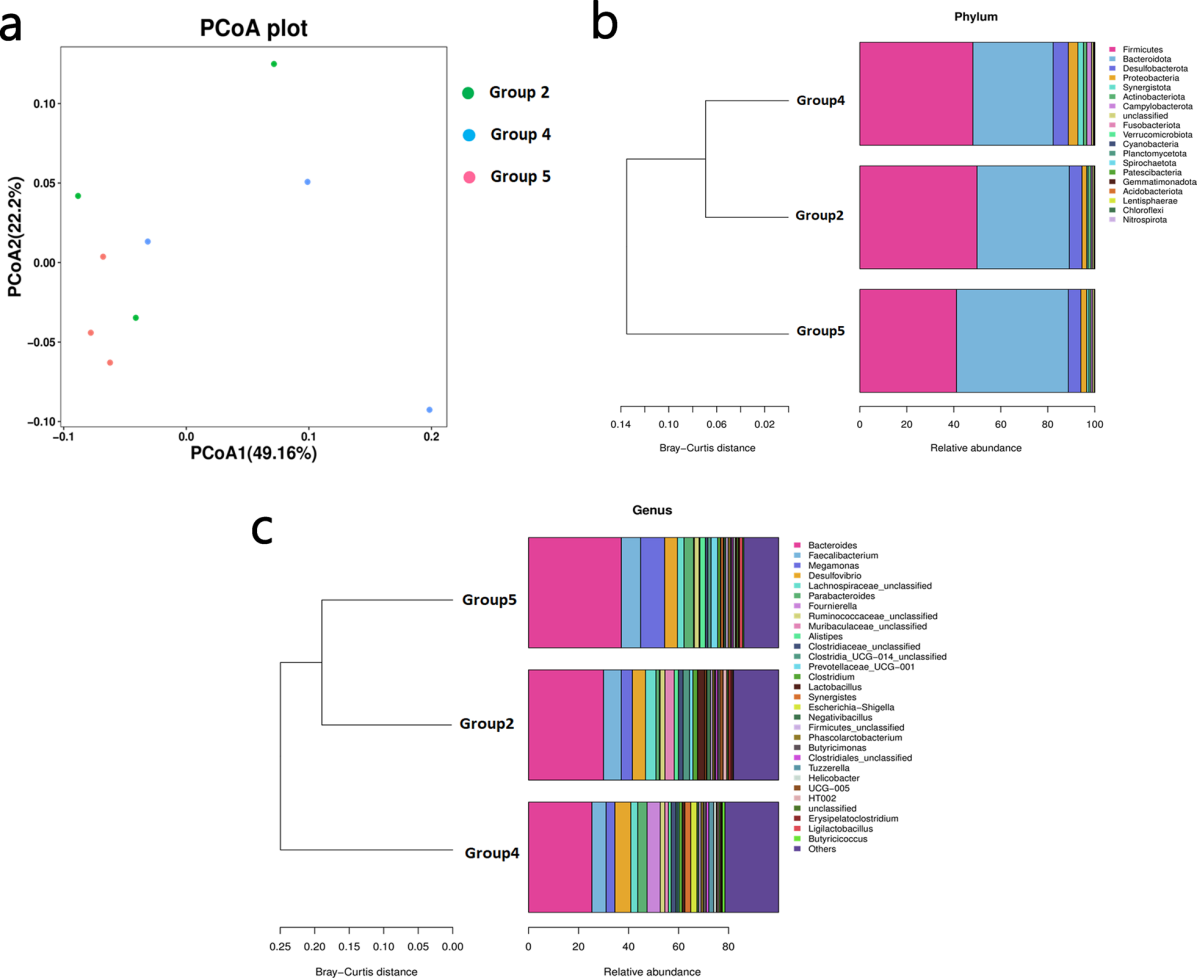
**a**–**c** Intestinal microbiota dysbiosis in the groups infected with *Eimeria tenella* and treated with GTPs. **a** Principal co-ordinates analysis (*PCoA*) plot of the cecal microbiota of the different groups. Relative abundance (%) of the bacteria at the phylum (**b**) and genus level (**c**)

The Shannon and Simpson alpha diversity indexes of the chicken gut microbiota were lowest for group 5 (control group) (Table 2). The highest Shannon and Chao indexes were for group 2 (ET + GTP group). The Simpson index was lower for group 2 than for group 4 (ET group). There was a clear distinction between the alpha diversity of the gut microbiota of the different groups (Fig. 6a).

**Table 2:** Alpha diversity indexes of the cecal microbiota in the different groups

Group	Shannon	Simpson	Chao
Group 2 (ET + GTP group)	7.030	0.975	610.677
Group 4 (ET group)	6.989	0.980	537.280
Group 5 (control group)	6.666	0.965	605.277

*ET* *Eimeria tenella* infection

At the phylum level, Firmicutes, Bacteroidota, Desulfobacterota and Proteobacteria were the most abundant in the cecal microflora (Fig. 6b). The abundance of Firmicutes in group 2, group 4 and group 5 was 49.88%, 48.13% and 41.13%, respectively. The abundance of Bacteroidetes was lower in group 4 (34.12%) than in group 2 (39.25%) and group 5 (47.58%). However, group 4 had higher abundances of Desulfobacterota (6.50%) and Proteobacteria (4.05%) than group 2 (5.41% and 1.97%, respectively) and group 5 (5.36% and 2.49%, respectively). The 30 most abundant genera of the cecal microflora are shown in Fig. 6c. Group 4 had lower proportions of *Bacteroides* (25.33%), *Faecalibacterium* (5.70%), *Megamonas* (3.50%), *Ruminococcaceae*_unclassified (1.84%), *Alistipes* (1.08%) *Clostridia*_UCG-014_ unclassified (0.62%) and *Prevotellaceae*_UCG-001 (0.56%) than group 2 (29.96%, 7.11%, 4.46%, 1.94%, 1.68%, 2.59% and 1.33%, respectively) and group 5 (37.09%, 7.72%, 9.60%, 1.89%, 2.18%, 1.32% and 2.61%, respectively). The proportions of *Desulfovibrio* (6.38%), *Fournierella* (5.25%) and *Clostridiaceae*_unclassified (1.94%) were higher in group 4 than in group 2 (5.25%, 0.31% and 1.87%, respectively) and group 5 (5.13%, 0.32% and 0.96%, respectively). The proportions of *Lachnospiraceae*_unclassified (4.18%), *Muribaculaceae*_unclassified (3.70%), *Clostridium* (1.86%) and *Lactobacillus* (2.87%) were higher in group 2 than in group 4 (2.75%, 1.44%, 1.34% and 1.04%, respectively) and group 5 (2.65%, 0.35%, 1.12% and 0.20%, respectively). The abundance of *Parabacteroides* was higher in group 5 (3.79%) than in group 2 (1.33%) and group 4 (3.75%).

**Fig. 7:**
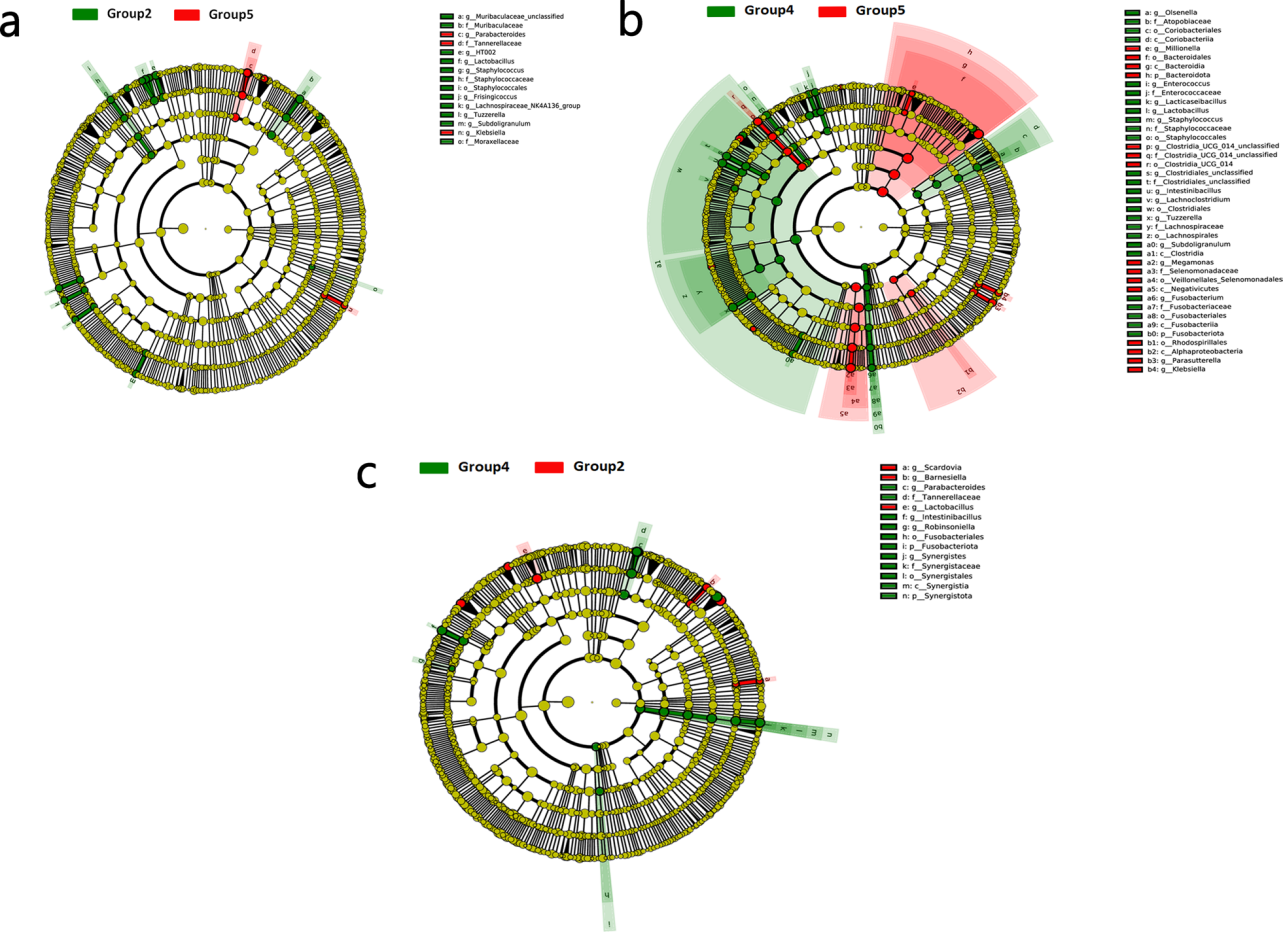
**a**–**c** Cladogram based on significant differences in the microbial flora of the treatment and control groups

At phylum level, the cladogram generated from the linear discriminant analysis effect size scores (Fig. 7) indicated significant differences between group 4 (ET group) and group 5 (control group) with respect to Bacteroidota and Fusobacteriota. There were also substantial differences between group 2 (ET + GTP group) and group 4 (ET group) with respect to Fusobacteriota and Synergistota. There were no differences between the gut microbiota of group 2 (ET + GTP group) and group 5 (control group) at the phylum level. The most significant differences in the cecal microflora at the genus level shown by the linear discriminant analysis effect size scores were between group 4 (ET group) and group 5 (control group).

### Predicted herbs targeting MMP1, MMP7, NOS2 and EPHA2

**Fig. 8:**
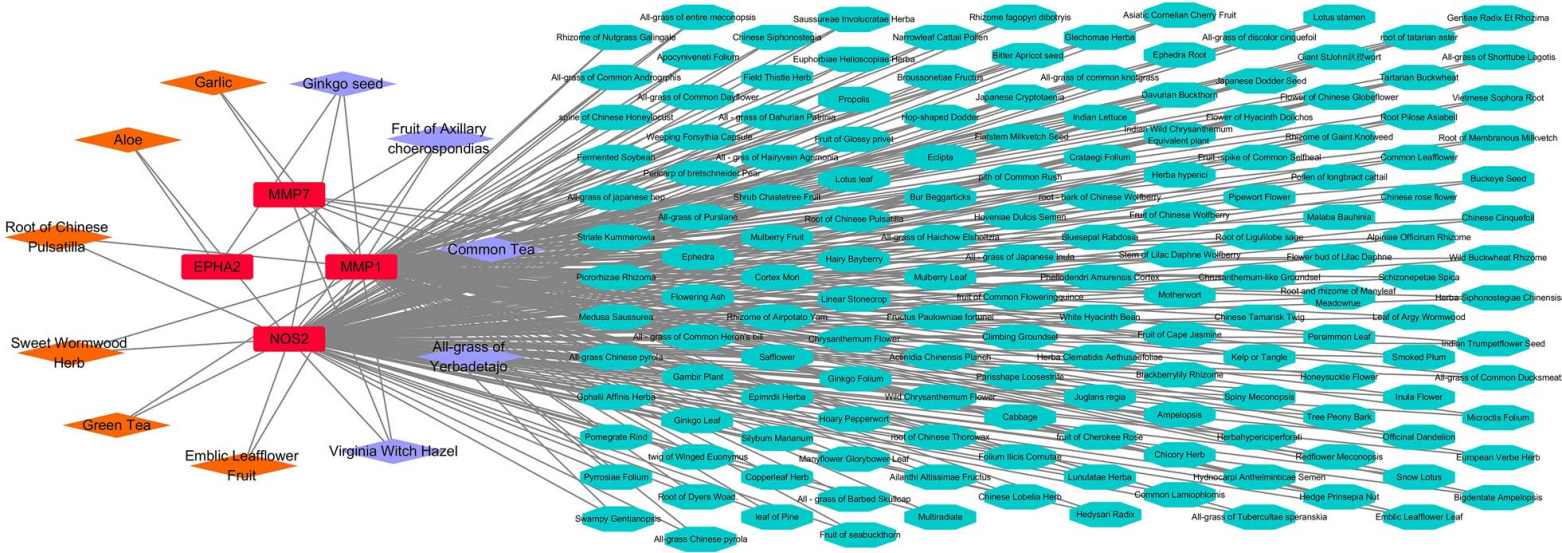
Predicted herb-target network. Red boxes indicate targeted proteins, orange diamonds herbs for which a regulatory relationship with the targets has been reported, purple diamonds herbs that have a regulatory effect on three targets, cyan ellipses herbs that have a regulatory effect on two targets

Herbs that target MMP1, MMP7, NOS2 and EPHA2 were predicted by using the HERB database; the 171 predicted herbs are listed in Additional file 2: Table S2. The herbs that were predicted to act on two or three targets were used to construct the herb-target network (Fig. 8). Ginkgo seed and fruit of Axillary choerospondias were predicted to interact with MMP1, NOS2 and EPHA2; all-grass of Yerbadetajo, common tea and Virginia witch hazel with MMP1, MMP7 and NOS2; aloe with EPHA2 and NOS2; and hoary pepperwort, garlic, hairy bayberry and kelp with MMP1 and MMP7. A total of 161 herbs were predicted to interact with MMP1 and NOS2, such as green tea, root of Chinese Pulsatilla and sweet wormwood.

## Discussion

The total cost of prophylaxis and chemotherapy for coccidiosis amounts to billions of pounds annually and is a huge burden for the poultry breeding industry [3]. Chemical drugs have been widely used for the treatment of coccidiosis in the poultry breeding industry; however, the extensive use of commonly used chemical drugs has led to the development of anticoccidial drug resistance [4, 37]. Therefore, it is thought necessary to identify and develop natural products or their derivatives as replacements for these commonly used drugs.

EGCG is the most abundant component (> 66%) of green tea polyphenols, followed by ECG, EC, and EGC [7]. Green tea and green tea byproducts have been widely utilized in the prevention and treatment of livestock diseases [17], as plant feed additives to improve the quality of livestock products [38] with respect to their nutritional effects [17], bacterial inhibition [10], activity against parasitic and viral infections [39, 40].

All of the chickens in the present study treated with green tea polyphenols experienced lesser symptoms in response to *E. tenella* infection (Table 1). ACI was highest in the chickens treated with 350 mg GTPs kg^−1^ body weight, and the ACI of group 2 was similar to that of group 3; in contrast, the efficacy of the GTPs was lowest for group 1, which was treated with 50 mg GTPs kg^−1^ body weight. This latter, relatively low dose of green tea polyphenols had a limited anticoccidial effect, but the highest dose did not significantly increase the anticoccidial effectivity in comparison to the medium dose. Thus, the medium dose is considered to be the most cost-effective one.

The cecal transcriptome of the chickens infected with *E. tenella* was investigated to determine changes in the expression of specific genes following their treatment with green tea polyphenols. In accordance with the ACI indexes, we examined the cecal transcriptome of group 2 (ET + GTP group), group 4 (ET group) and group 5 (control group) by RNA-seq, and validated the DEGs by qPCR (Fig. 5). Group 4 (ET group) had the largest number of differentially upregulated and downregulated genes compared with group 5 (control group), followed by group 2 (ET + GTP group). The smallest differences in upregulated and downregulated genes were between group 2 (ET + GTP group) and group 4 (ET group) (Fig. 1a). There were nine DEGs (*MMP7*,* NOS2*,* EPHA2*,* SPP1*, cell wall biogenesis 43 C-terminal homolog,* MMP1*,* DUSP6*, ENSGALG00000051623 and* KCNJ5*) common to groups 2, 4 and 5 (Fig. 1b). The results indicated that *E. tenella* interfered with and tea polyphenols regulated these genes. The three most upregulated genes in group 4 (ET group) compared with group 5 (control group) were* MMP1*,* MMP7* and ENSGALG00000025899. However, all of these genes were significantly downregulated in group 2 (ET + GTP group) compared with group 4 (ET group). The metrics for the clustering of the common genes was similar for group 2 (ET + GTP group) and group 5 (control group) (Fig. 1c). These results indicate that the green tea polyphenols tested here may reduce the impact of *E. tenella* infection on the expression of these genes.

Eight genes (*MMP7*,* MMP1*,* NOS2*,* EPHA2*, sterile alpha motif domain containing 9 like, coagulation factor X,* IFIH1* and hepatocyte growth factor) were commonly predicted target genes (Fig. 2a). The compound-target-KEGG network diagram (Fig. 2c) indicated that EC, EGC and EGCG had more of an effect on the ecpression of these genes than ECG and GCG. EGCG in particular had an effect on all eight genes. The molecular docking results also showed high binding affinity between EGCG and IFIH1 (Fig. 2d). These results demonstrated that EGCG played an important role in the anticoccidial process in the chickens.

MMPs are a family of zinc-dependent endopeptidases that play important roles in physiological processes and pathological conditions, and are involved in the degradation of various proteins in the extracellular matrix [41]. In this study, several MMP genes, including* MMP1*,* MMP7*,* MMP9*,* MMP10* and* MMP27*, were upregulated post *E. tenella* infection, which is consistent with a previous report [42]. MMPs, which are associated with processes of tissue remodeling, are also expressed in other protozoan parasitic infections, such as with *Plasmodium*, *Trypanosoma brucei*, *Leishmania* and *Toxoplasma gondii* [43]. EGCG is the most abundant component of green tea polyphenols that can decrease the expression of* MMP2*,* MMP9* [44] and* MMP7* [6]. In the present study, the expression of* MMP1* and* MMP7* was downregulated in group 2 (ET + GTP group) compared to group 4 (ET group). These results indicate that these MMP genes may be the targets of green tea polyphenols, which may in turn explain why treatment with the latter contributes to the resistance of chickens to *E. tenella* infection.

NOS2 is the principal enzyme in the production of NO [45], which plays an important role in defense against infectious organisms. During *E. tenella* infection, the level of NO is increased to reduce the damage caused by this parasite [46, 47]. NOS2 was significantly upregulated in group 4 compared with group 5 [log2(fc) = 3.52, *q* < 0.01], significantly downregulated in group 2 compared with group 4 [log2(fc) = − 2.19, *q* < 0.01], and significantly upregulated in group 2 compared with group 5 [log2(fc) = 1.33, *q* < 0.01]. Various inflammatory cytokine genes, including those coding for IL-8 and IL1B, were significantly upregulated (*q* < 0.01) in group 4 compared with group 5. The genes were slightly downregulated in group 2 compared with group 4, though the difference was not statistically significant (*q* > 0.05). The excessive production of NO, IL-8 and IL1B is harmful to the host [48, 49]. Previous studies have reported that green tea and green tea polyphenols can scavenge NO [11]. This suggests that, in the present study, the treatment of the chickens with green tea polyphenols may have reduced an excessive inflammatory response caused by NO during their infection with *E. tenella*.

DUSP6 is a member of the MAPKs phosphatase family, which regulate cell proliferation, growth and survival in physiological or pathological conditions. DUSPs have a negative regulatory effect on MAPKs [50], and inhibition of the MAPK pathway could decrease cell invasion by *E. tenella* [51]. DUSP4 plays a protective role in *T. gondii* and *Leishmania mexicana* infections [52]. In this study, the upregulation of* DUSP4* and* DUSP6* in group 4 (ET group) compared with group 5 (control group) may indicate the positive regulation of DUSPs in *E. tenella* infection.

*Eimeria* spp. are highly successful obligate intracellular parasites and can cause damage to host cells through excessive oxidative stress [53]. GO and KEGG analyses are helpful for understanding the primary functions of DEGs. GO analysis showed that the DEGs were commonly enriched in oxidation–reduction processes (Fig. 3). In the comparison of group 4 (ET group) with group 5 (control group), 46 DEGs were enriched in oxidation–reduction process, while only 11 DEGs were enriched in this process in group 2 (ET + GTP group) compared with group 5 (control group), and 10 DEGs were enriched in this process in group 2 (ET + GTP group) compared with group 4 (ET group). This indicates that green tea polyphenols play a role as antioxidants [8, 54] in response to the infection process. GO analysis showed that integral component of membrane had 65 DEGs, 165 DEGs and 28 DEGs in group 2 (ET + GTP group) compared with group 5 (control group), group 4 (ET group) compared with group 5 (control group) and group 2 (ET + GTP group) compared with group 4 (ET group), respectively. The lower number of DEGs indicated that treatment with green tea polyphenols had an effect on the response of the host cells to the parasitic invasion. In comparison with group 4 (ET group) and group 5 (control group), the green tea polyphenol treatment in group 2 (Fig. 4) may have enriched DEGs in the phagosome pathway that produces superoxide.

Reports on shifts in the relative abundance of intestinal microflora during *E. tenella* infection are inconsistent. In the present study, the microbial diversity was increased post *E. tenella* infection (Table 2), and the trends of variation in the Shannon, Simpson and Chao indexes are similar to those of a previous study [55], although different from those reported by Huang et al. [56, 57]. The number of sporulated oocysts, age of the chickens, breed of the chickens and duration of *E. tenella* infection may have contributed to these differences.

As shown in Fig. 6, the intestinal flora differed between the three groups treated with green tea polyphenols. The relative abundance of Firmicutes and Proteobacteria was increased, whereas that of Bacteroidetes was decreased in group 4 (ET group) compared with group 5 (control group). These results differ from those of a recent study [55], although they are similar to those of Zhou et al. [56]. At the genus level, the abundances of *Bacteroides*, *Faecalibacterium*, *Megamonas*, *Alistipes* and *Prevotellaceae*_UCG-001 were reduced in group 2 (ET + GTP group) compared with group 5 (control group) and in group 4 (ET group) compared with group 5 (control group), but were higher in group 2 (ET + GTP group) than in group 4 (ET group). The most notable difference between the intestinal flora was between group 4 (ET group) and group 5 (control group) (Fig. 7). These results indicate that *E. tenella* infection can modulate the abundance of these microbes, and that the tea polyphenols can mitigate this effect.

The antibacterial mechanism of action of green tea polyphenols is complicated. It is clear that they can form hydrogen bonds and show hydrophobic interaction with the proteins or DNA of bacteria, and therefore inhibit their replication [10]. Previous studies have demonstrated that probiotics can regulate the expression of host genes involved in immunity, gut barrier integrity, homeostasis and metabolism [58–60]. In this study, the abundance of *Lactobacillus* was higher in group 2 (ET + GTP group) than in group 4 (ET group) and group 5 (control group) (Fig. 7). *Lactobacillus* is a probiotic which is beneficial to the host’s immunoregulation, antipathogenic mechanisms and intestinal epithelial barrier [61, 62]. *Lactobacillus* has been proven to be an effective anticoccidial microbe [63, 64], thus the enrichment in probiotics of the groups treated with green tea polyphenols suggests that these compounds can enhance the resistance of chickens against *E. tenella* infection through their positive effect on the levels of these microorganisms.

There are three developmental stages in the life cycle of *E. tenella*: sporogony, merogony and gametogony [42, 65], with the latter two stages localized in the epithelial cells of the chicken cecum. The specific host-*E. tenella* interaction involves changes in the expression of a large number of host genes [66, 67], and the treatment of this complex disease via the simultaneous treatment of multiple targets was found to be more effective than treating a single target [68]. Chinese medicinal herbs contain multiple bioactive components which have multiple targets [27, 69]. They have multiple therapeutic effects in the treatment of complex parasitic diseases through the synergistic effects of multiple components on multiple targets. Because MMP7, MMP1, NOS2 and EPHA2 were found in the same samples (Figs. 1b, 2a), we suggest that the predicted herbs can act on two or three of these targets. Among the predicted herbs (Fig. 8), anticoccidial effects of garlic, emblic leafflower fruit, green tea, root of Chinese pulsatilla, aloe and sweet wormwood have been reported [70, 71]. These results indicate that network pharmacology is an effective method for searching for novel drugs against parasitic diseases. Furthermore, another 165 herbs predicted in this study could be used to compose a pool of Chinese medicinal herbs for the development of anticoccidial drugs, which could contribute to the control of drug resistance in *Eimeria* parasites.

**Fig. 9:**
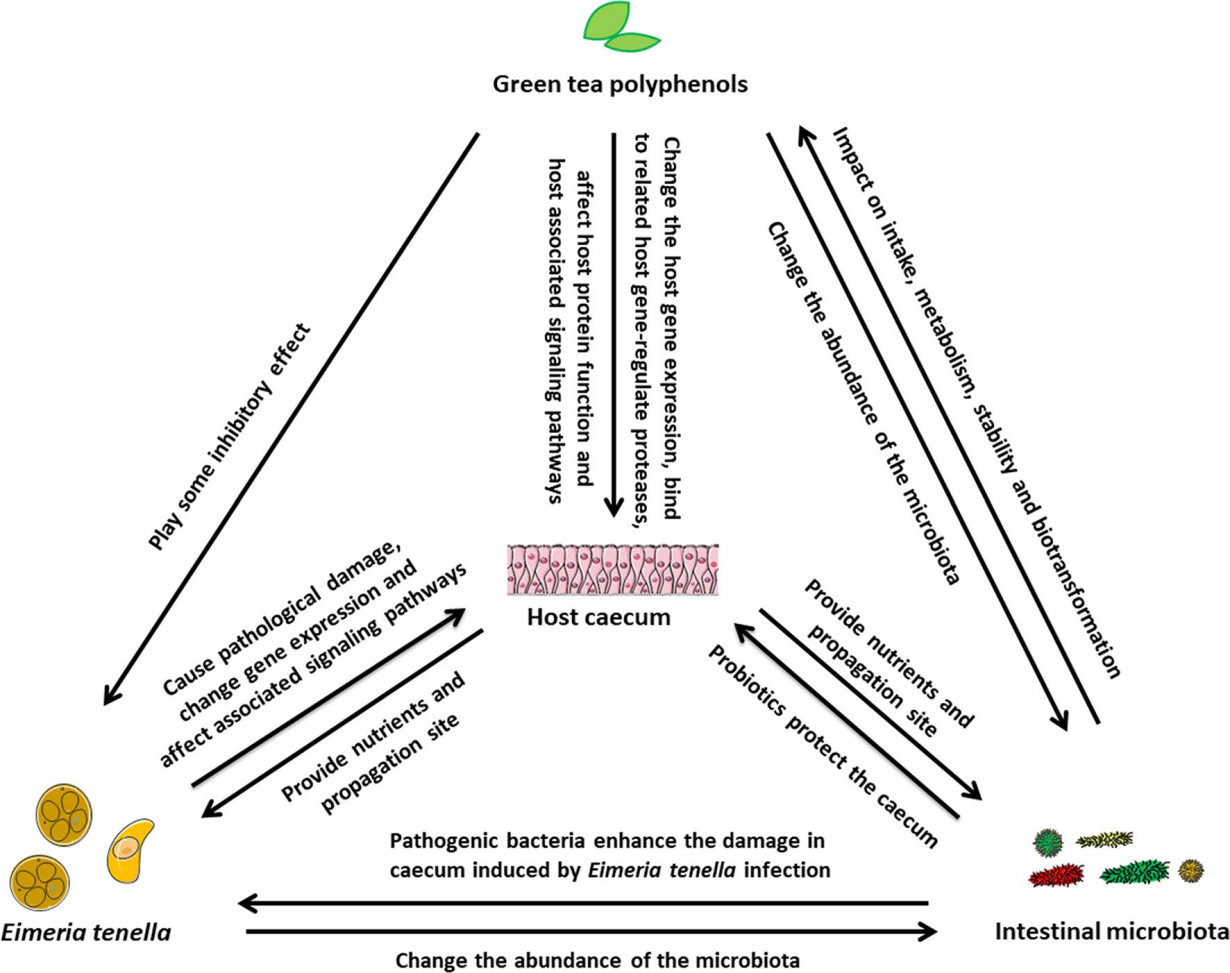
Potential relationships between green tea polyphenols, *Eimeria tenella,* host cecum and the host intestinal microbiota

The anticoccidial effects of green tea polyphenols in Wuliangshan black-boned chickens infected with *E. tenella* infection are thought to be multiple. Green tea polyphenols may have direct or indirect effects on the host, its microbiota, and *E. tenella*, as indicated in Fig. 9. For example, *E. tenella* infection of the host cecum, which provides nutrients and propagation sites for this parasite, induces tissue damage and changes in gene expression, and treatment with green tea phenols may have an effect on host gene expression, host protein function and host immunity-associated signaling pathways that could inhibit this parasite. It is also possible that pathogenic bacteria in the host intestine could enhance the damage to the cecum induced by *E. tenella* infection, and that treatment with green tea polyphenols could mitigate this by changing the abundance of some of the host microbiota including that of probiotics (e.g. *Lactobacillus*), which could reduce this damage through strengthening of the gut mucosal barrier, physical exclusion of pathogens, production of antimicrobial substances and modulation of the immune system [72]. Additionally, the host microbiota has an impact on the uptake, metabolism, stability and biotransformation of green tea polyphenols [73]. With respect to future practical applications of tea polyphenols, such as feed additives, as the chickens in this study were treated with these for their entire feeding cycle, i.e. including before *E. tenella* infection (8–20 days old) and after *E. tenella* infection (21–28 days old), it is possible that they have both a prophylactic and treatment effect. A limitation of this study is that we were unable to distinguish prophylaxis from treatment effects of the green tea polyphenols with respect to *E. tenella* infection in the chickens; hence, further studies are needed to achieve this.

## Conclusions

RNA-seq, network pharmacology and 16S rRNA sequencing revealed how green tea polyphenols regulate the gene expression and gut microbiota of chickens during *E. tenella* infection.* MMP7*,* MMP1*,* NOS2* and* EPHA2*, and probiotics (*Lactobacillus*), are potential candidates for further investigation as anticoccidials. EGCG, the main component tea polyphenol, plays an important role in the anticoccidial process. The green tea polyphenols tested here and some of the herbs of our predicted Chinese medicinal herb pool are candidate anticoccidial feed additives that may offer a solution to the problem of anticoccidial drug resistance in chickens.

### Supplementary Information

**Additional file 1: Table S1.** The primers used in the qPCR experiment.

**Additional file 2: Table S2. **The green tea polyphenol-associated targets obtained from the HERB, TCMSP and PharmMapper databases are shown in sheet 1, and predicted herbs targeting MMP1, MMP7, NOS2 and EPHA2 from the HERB database are shown in sheet 2.

## Data Availability

The original datasets of the present study have been submitted to the National Centre for Biotechnology Information. The bio-project number PRJNA951256 is for the transcriptomic data and PRJNA952316 is for the 16S rRNA data.

## Abbreviations

ACI: Anticoccidial index
DEGs: Differentially expressed genes
DUSP: Dual-specificity phosphatase
EC: (-)-Epicatechin
ECG: (-)-Epicatechin gallate
EGC: (-)-Epigallocatechin
EGCG: (-)-Epigallocatechin gallate
EPHA2: Ephrin type-A receptor 2
GCG: (-)-Gallocatechin gallate
GO: Gene Ontology
IFIH1: Interferon induced with helicase C domain 1
IL-8: Interleukin 8
IL1B: Interleukin 1 beta
KEGG: Kyoto Encyclopedia of Genes and Genomes
MAPK: Mitogen-activated protein kinase
MMP: Matrix metalloproteinase
NOS2: Nitric oxide synthase 2
PCR: Polymerase chain reaction
qPCR: Quantitative real-time polymerase chain reaction
RNA: Ribonucleic acid
RNA-seq: Ribonucleic acid sequencing
16S rRNA: 16S ribosomal ribonucleic acid
SPP1: Secreted phosphoprotein 1
TCMSP: Traditional Chinese Medicine Systems Pharmacology Database and Analysis Platform
